# Saturated Solution of Corrosive Sublimate in Erysipelas

**Published:** 1843-01

**Authors:** 


### SATURATED SOLUTION OF CORROSIVE SUBLIMATE IN ERYSIPELAS.

By a mistake, in preparing the index to our last valume, a valuable paper on this subject by Dr. Tripler, an army surgeon, was omitted; and therefore will not, we fear, receive the attention it merits. When in Detroit, the Doctor assured us of the great efficacy of this solution, acknowledging himself indebted for the recipe, to the very respectable Dr. Pitcher, formerly of the army, but now a resident physician in that city. A scruple of the sublimate is dissolved in an ounce of rain or distilled water, and lint dipped in it is laid over the inflamed part, being extended a short distance upon the sound. It gives no pain, and arrests the inflammation with great promptness. Doctor T. has chiefly used it in the erysipelas of the scalp and face of soldiers, a frequent disease in the Detroit barracks, and in no case, as we understood him, did the malady subsequently attack the brain. We would, however, refer our readers to his paper in the last number of our last volume, to do which is the special object of this article. D.

